# Cerebrospinal Fluid Metabolome in Central Nervous System Infections: A Study of Diagnostic Accuracy

**DOI:** 10.1002/ana.27291

**Published:** 2025-06-17

**Authors:** Steven L. Staal, Sabine E. Olie, Michel van Weeghel, Bauke V. Schomakers, Frédéric M. Vaz, I‐PACE Study Group, Diederik van de Beek, Matthijs C. Brouwer

**Affiliations:** ^1^ Amsterdam UMC University of Amsterdam, Department of Neurology, Amsterdam Neuroscience Amsterdam the Netherlands; ^2^ Amsterdam UMC, University of Amsterdam, Core Facility Metabolomics Amsterdam the Netherlands; ^3^ Amsterdam UMC, Department of Laboratory Medicine and Pediatrics, Laboratory Genetic Metabolic Diseases, Emma Children's Hospital University of Amsterdam Amsterdam the Netherlands; ^4^ Amsterdam Gastroenterology Endocrinology Metabolism, Inborn Errors of Metabolism Amsterdam the Netherlands

## Abstract

**Objective:**

To assess the diagnostic accuracy of metabolites in cerebrospinal fluid (CSF) for central nervous system (CNS) infections.

**Methods:**

Patients were derived from three prospective cohort studies in the Netherlands. All studies included adults suspected of a CNS infection who underwent a diagnostic lumbar puncture. Metabolomics was performed on CSF using ultra‐high‐performance liquid chromatography with tandem mass spectrometry on a discovery and validation cohort. Metabolite quantification was the index test; a microbiologically confirmed diagnosis was the reference standard.

**Results:**

In total, 343 episodes were included, of whom 170 (50%) had a CNS infections and 173 (50%) episodes had other diagnoses. CNS infections included bacterial meningitis in 88 (26%), viral meningoencephalitis in 50 (15%), and other CNS infections in 32 (9%) episodes. Other diagnoses consisted of CNS autoimmune disorders in 21 (6%), other neurological diseases in 84 (24%), and systemic infections in 68 (20%) episodes. A distinct metabolomic profile was observed in CSF of CNS infections, particularly bacterial meningitis. Glucose, glycerate, 1.3‐diphosphoglyceric acid, pyruvate, lactate, taurine, and alpha‐ketoglutarate had the highest diagnostic accuracy (area under the curve 0.87 to 0.95). Combinations further improved diagnostic accuracy, resulting in models that outperformed both individual metabolites and CSF leukocytes. Episodes with CSF leukocytes between 5 and 1,000 cells per mm^3^ showed similar results.

**Interpretation:**

CSF metabolites demonstrate high diagnostic accuracy for CNS infections, particularly bacterial meningitis. Combinations further improve the diagnostic performance, exceeding that of CSF leukocytes alone. These findings highlight the potential of cerebrospinal fluid metabolites to improve diagnostic accuracy in clinical practice. ANN NEUROL 2025;98:851–863

Central nervous system (CNS) infections are a medical emergency that require rapid diagnosis and immediate treatment. Currently, the best predictor for a CNS infection is an elevated cerebrospinal fluid (CSF) leukocyte count, but this is also found in approximately half of the patients without a CNS infection.^1^ In addition, CSF leukocytes might sometimes even be normal.^2^, ^3^ Detecting the causative microorganism in CSF through culture or nucleic acid amplification tests is the gold standard for CNS infections.^4^ However, CSF cultures take time to become positive, and the yield depends on the causative microorganism and antibiotic pretreatment, whereas polymerase chain reaction (PCR) is specific only to the tested pathogens.^4^, ^5^ Furthermore, a negative CSF culture or PCR does not rule out a CNS infection. Hence, there is a need for new diagnostic markers for early differentiation between CNS infections and non‐CNS infections.

Metabolomics is a phenotyping technique that comprises of a targeted and untargeted approach to identify and quantify the metabolite profile in a specimen. As such, it renders a temporal fingerprint of the result of all metabolic processes in a sample. Untargeted metabolomics has provided detailed information on metabolic pathways in a variety of neurological diseases, and has the potential to discover yet unexplored disease‐specific metabolite alterations that could serve as potential diagnostic biomarkers.^6^, ^7^, ^8^ However, little is known about the diagnostic accuracy of CSF metabolites in CNS infections, as previous studies are often limited by small sample sizes, targeted analyses rather than an untargeted approach or comparisons focused on specific CNS infections instead of assessing them in the context of all patients with suspected CNS infections. We performed untargeted metabolomics on CSF from patients with suspected CNS infections to identify metabolites that could serve as potential biomarkers for diagnosing or distinguishing different kinds of CNS infections.

## Methods

### Study Population and Data Collection

For this study we included adult patients from 3 prospective cohort studies, the PACEM (Pediatric and Adult Causes of Encephalitis and Meningitis) study, the I‐PACE (Improving Prognosis by Using Innovative Methods to Diagnose Causes of Encephalitis) study, and the MeninGene study.^1^, ^9^, ^10^ The PACEM study (2012–2015) served as a single‐center pilot study for the ongoing multicenter cohort study, the I‐PACE study, which started in 2017. Detailed information on the methods and the study population have been described previously.^1^, ^10^ Both studies were conducted in the Netherlands, and included patients aged ≥16 years, suspected of a CNS infection, who underwent a diagnostic lumbar puncture. The MeninGene study started in 2006, and is an ongoing nationwide cohort study of community‐acquired bacterial meningitis in the Netherlands.^9^ Briefly, patients aged ≥16 years were included with either a positive CSF culture or a positive CSF bacterial antigen test, a positive bacterial CSF PCR and/or a positive blood culture in combination with an individual CSF predictor for bacterial meningitis (CSF leukocytes >2,000 cells/mm^3^, CSF granulocytes >1,180 cells/mm^3^, glucose <1.9 mmol/L, CSF serum glucose ratio <0.23, or protein concentration >2.2 gram/L).^11^ Exclusion criteria were similar across all studies, and consisted of neurosurgery or severe head trauma (1–3 months) prior to the lumbar puncture, the presence of a neurosurgical or neurostimulator device, or a diagnosis of hospital‐acquired bacterial meningitis (defined as bacterial meningitis occurring during admission or within 1 week after discharge).

The discovery cohort consisted of all consecutive patients from the PACEM study of whom sufficient CSF was available. Additional patients with community‐acquired bacterial meningitis were derived from the MeninGene study through convenience sampling to approximate actual disease prevalence, and were included between 2012 and 2019. The validation cohort consisted of randomly selected patients from the I‐PACE study, included between 2017 and 2023, and was again supplemented with community‐acquired bacterial meningitis patients from the MeninGene study, included between 2006 and 2023. This study was reported in accordance with the Standards for Reporting Diagnostic accuracy studies (STARD) reporting guideline.^12^

All studies were conducted in the Netherlands, and written informed consent was obtained from the patient or their legal representative. Anonymized clinical data were collected in secured online case record forms. All studies were approved by the medical research or biobank ethics committee of the Amsterdam UMC, Amsterdam, the Netherlands (number: AMC2013_043 [MeninGene] and AMC2014_290 [PACEM/ I‐PACE]).

### Diagnostic Categories and Comparisons

Clinical diagnoses were used as the reference standard, and were divided over the following prespecified categories, as previously described^1^: CNS infection, CNS autoimmune disorder, other neurological disease, and systemic disease. CNS infections were subdivided into bacterial meningitis, viral meningoencephalitis, and other CNS infection. All episodes were independently evaluated and categorized by two clinicians (S.S. and S.O.); disagreements between the clinicians were resolved by the decision of a third clinician (M.B.), a neurologist specialized in CNS infections and inflammatory disorders. The definition and rationale of the diagnostic classifications and their use as the reference standard have been described previously.^1^ Clinical diagnoses were based on all available clinical information, including clinical symptoms, physical examination, and ancillary investigation. No information on the reference standard was available to the performer of the index test, and the metabolomics assays were performed by technicians that did not have access to the clinical information.

Comparisons were made between the following diagnostic (sub‐)categories to best reflect diagnostic considerations in clinical care settings: (1) CNS infection versus other diagnoses, (2) bacterial meningitis versus all other diagnoses, and (3) bacterial meningitis versus viral meningoencephalitis. Analyses were initially performed on microbiologically confirmed episodes only. Subsequently, the analyses were performed in the entire study population (including episodes with and without microbiological confirmation) and in patients with CSF leukocytes between 5 and 1,000/mm^3^. The rationale for the latter was that the diagnostic uncertainty is highest within this patient population.^13^ The results from the discovery cohort were validated in the validation cohort.

### Sample Collection and Metabolomics

Metabolomics, the index test, was performed on residual CSF from the initial diagnostic lumbar puncture. Residual CSF was stored in a central biobank in Amsterdam UMC at −80°C. The method for metabolomics was tailored specifically for high confidence analyses of key polar metabolites of the most fundamental metabolic pathways, instead of a broad untargeted approach in which identification of metabolites is less certain. Metabolomics was performed as previously described,^14^, ^15^ with minor adjustments. A 75‐μL mixture of the following internal standards in water was added to each CSF sample (100 μL): adenosine‐^15^N_5_‐monophosphate (100 μM), adenosine‐^15^N_5_‐triphosphate (1 mM), D_4_‐alanine (100 μM), D_7_‐arginine (100 μM), D_3_‐aspartic acid (100 μM), D_3_‐carnitine (100 μM), D_4_‐citric acid (100 μM), ^13^C_1_‐citrulline (100 μM), ^13^C_6_‐fructose‐1,6‐diphosphate (100 μM), guanosine‐^15^N_5_‐monophosphate (100 μM), guanosine‐^15^N_5_‐triphosphate (1 mM), ^13^C_6_‐glucose (1 mM), ^13^C_6_‐glucose‐6‐phosphate (100 μM), D_3_‐glutamic acid (100 μM), D_5_‐glutaminutese (100 μM), ^13^C_6_‐isoleucine (100 μM), D_3_‐leucine (100 μM), D_4_‐lysine (100 μM), D_3_‐methionine (100 μM), D_6_‐ornithine (100 μM), D_5_‐phenylalanine (100 μM), D_7_‐proline (100 μM), ^13^C_3_‐pyruvate (100 μM), D_3_‐serine (100 μM), D_5_‐tryptophan (100 μM), D_4_‐tyrosine (100 μM), and D_8_‐valine (100 μM). Subsequently, 325 μL water, 500 μL methanol, and 1 mL chloroform were added and thoroughly mixed before centrifugation for 10 minutes at 20.000 g. The top layer, containing the polar phase, was transferred to a new 1.5‐mL tube and dried using a vacuum concentrator at 60°C. The dried samples were then reconstituted in 100 μL methanol/water (6/4; v/v). Metabolites were analyzed using a Waters Acquity ultra‐high‐performance liquid chromatography system (Milford, MA, USA) coupled to a Bruker Impact II™ Ultra‐High Resolution Qq‐Time‐Of‐Flight mass spectrometer (Billerica, MA, USA). Of each sample, 5 μL was injected and kept at 12°C during analysis. Chromatographic separation was achieved by using a Merck Millipore SeQuant ZIC‐cHILIC column (Burlington, MA, USA; PEEK 100 × 2.1 mm, 3 μm particle size) at 30°C. Mobile phase consisted of (1) 1:9 acetonitrile : water, and (2) 9:1 acetonitrile : water, both containing 5 mM ammonium acetate. Using a flow rate of 0.25 mL/min, the liquid chromatography gradient consisted of: Dwell at 100% Solvent B, 0–2 minutes; Ramp to 54% Solvent B at 13.5 minutes; Ramp to 0% Solvent B at 13.51 minutes; Dwell at 0% Solvent B, 13.51–19 minutes; Ramp to 100% B at 19.01 minutes; and Dwell at 100% Solvent B, 19.01–19.5 minutes. Column equilibration was achieved by increasing the flow rate to 0.4 mL/min at 100% Solvent B from 19.5 to 21 minutes. Mass spectrometer data were acquired using negative and positive ionization in full scan mode over the range of *m/z* 50–1,200. Data were analyzed using Bruker TASQ software version 2021b (2021.1.2.452). Metabolite intensities were normalized to dry tissue weight, as well as to internal standards with comparable retention times and response in the mass spectrometer. Metabolite identification was based on a combination of accurate mass, (relative) retention times and fragmentation spectra, compared with the analysis of a library of standards. Quality control was performed by assessing the coefficient of variance percentage of each metabolite from the repeated measurements of a pooled sample. Metabolites with a coefficient of variance percentage ≥25% were considered to have high variance across samples (ie, less stable measurements) and should therefore be interpreted with caution. However, these metabolites were not removed from the analyses, because they may still provide valuable insight into biological processes.

### Statistical Analysis

Statistical analyses and data visualization were performed in RStudio version 4.3.2 (RStudio, Boston, MA, USA). As this study was considered exploratory and data on diagnostic accuracy of the index test were unavailable, a power calculation could not be performed. Missing metabolite values were replaced by one‐fifth of the lowest value of the corresponding metabolite; metabolites with missing values for >25% of samples were excluded. Normality of the data was evaluated by visual inspection of histograms and Q‐Q plots. A *p* value ≤0.05 was considered statistically significant. Where appropriate *p* values were adjusted for multiple testing using Benjamini–Hochberg's false discovery rate (FDR). Comparisons of individual metabolites between categories were performed using a Student *t* test for normally distributed data, and a Mann–Whitney *U* test for non‐normally distributed data; *p* values were adjusted for multiple testing. A summary of the relative differences was visualized by plotting the log^2^ (fold change) of either the mean or the median against the –log^10^ FDR using volcano plots, labeling the top 5 metabolites.

To assess and optimize the separation of CSF metabolome profiles between categories, we performed a partial least squares‐discriminant analysis on log_10_‐transformed, centered, and scaled data from the significantly altered metabolites (FDR <0.05) using the R package mixOmics (Toulouse, France).^16^ Variable importance in projection scores were assessed for each metabolite to evaluate individual contributions to the observed separation, and metabolites with a variable importance in projection score >1 were kept for further downstream analyses. Remaining metabolites were assessed for linearity between their respective diagnostic categories and, were appropriate, values underwent log^10^ or square root transformation. R‐package pROC^17^ (Geneva, Switzerland) was used to produce receiver operating characteristic curves to evaluate diagnostic accuracy by calculating the area under the curve (AUC) of linear metabolites. Here, CSF leukocytes, previously identified as the best predictor of both CNS infection and bacterial meningitis, was added for comparison.^1^ An AUC of >0.90 was considered excellent, 0.80–0.90 good, 70–80 fair, and <70 poor discrimination.

Finally, least absolute shrinkage and selection operator (LASSO) regression was applied to all linear metabolites, along with CSF leukocytes, to determine the best (combination of) variable(s) for predicting a diagnostic category. Selected metabolites were evaluated in a multivariable logistic regression model followed by backward stepwise selection. To reduce the risk of overfitting and to enhance the robustness of the selected variables, we repeated the aforementioned analysis in 100 bootstrap samples from the discovery dataset. Variables that were selected in >50% of the bootstrap samples were included in the final multivariable model. For each final model we calculated the sensitivity, specificity, positive predictive value, and negative predictive value, and we determine the Youden's index, a cut‐off value that maximizes the sensitivity and specificity. LASSO regression was performed using R‐packages glmnet and rms.^18^ Other R‐packages used were openxlsx, tidyverse, and reshape (for data handling), ggplot2 and ggrepel (for data visualization), and tableone (for data representation).

## Results

### Cohort Characteristics

In total, 343 episodes with a suspected CNS infection were included in this study. The discovery cohort consisted of 235 episodes (206 were from the PACEM study and 29 from the MeninGene study); the validation cohort consisted of 108 episodes (59 were from the I‐PACE study and 49 from the MeninGene study). The overall median age was 51 years (interquartile range [IQR] 35–64) and 182 of 343 (53%) episodes were women (Table 1). In the discovery cohort, a CNS infection was diagnosed in 82 of 235 (35%) episodes, and consisted of bacterial meningitis in 39 of 235 (17%) episodes, viral meningoencephalitis in 31 (13%), and other CNS infections in 12 (5%). Other diagnoses were CNS autoimmune disorders in 17 of 235 (7%) episodes, other neurological diseases in 74 (31%), and systemic diseases in 62 (26%). In the validation cohort, CNS infections were diagnosed in 88 of 108 (81%) episodes. Bacterial meningitis occurred in 49 of 108 (45%) episodes, viral meningoencephalitis and other CNS infections occurred in 19 (18%) and 20 (19%) episodes, respectively. Other diagnoses consisted of CNS autoimmune disorders in 4 of 108 (4%) episodes, other neurological diseases in 10 (9%), and systemic infections in 6 (6%).

**Table 1:** Demographics and Diagnoses per Study Cohort

	Overall (*n* = 343)	Derivation cohort (*N* = 235)		Validation cohort (*N* = 108)	
		PACEM (*n* = 206)	MeninGene (*n* = 29)	I‐PACE (*n* = 59)	MeninGene (*n* = 49)
Age (yr)	51 (35–64)	49 (34–64)	59 (33–68)	48 (35–63)	58 (45–66)
Sex (F)	182 (53%)	116 (56%)	17 (59%)	25 (42%)	24 (49%)
CNS infection	170 (50%)	53 (26%)	29 (100%)	39 (66%)	49 (100%)
Bacterial meningitis	88 (26%)	10 (5%)	29 (100%)		49 (100%)
* Confirmed in CSF	85 (25%)	7 (3%)	29 (100%)		49 (100%)
*H. influenzae*	2 (2%)				2 (4%)
*L. monocytogenes*	11 (13%)		5 (17%)		6 (12%)
*N. meningitidis*	19 (22%)	2 (29%)	9 (31%)		8 (16%)
*S. pneumoniae*	50 (59%)	3 (43%)	15 (52%)		32 (65%)
Others^a^	3 (4%)	2 (29%)			1 (2%)
Viral CNS infection	50 (15%)	31 (15%)		19 (32%)	
*Confirmed in CSF	37 (11%)	18 (9%)		19 (32%)	
Enterovirus	12 (32%)	4 (22%)		8 (42%)	
HSV‐1	2 (5%)	1 (6%)		1 (5%)	
HSV‐2	7 (19%)	2 (11%)		5 (26%)	
HSV unspecified	3 (8%)	2 (11%)		1 (5%)	
VZV	9 (24%)	5 (28%)		4 (21%)	
Others^b^	4 (11%)	4 (22%)			
Other CNS infection	32 (9%)	12 (6%)		20 (34%)	
* Confirmed in CSF	20 (6%)	9 (4%)		11 (19%)	
*A. cantonensis*	2 (10%)	2 (22%)			
*B. burgdorferi*	4 (20%)	1 (11%)		3 (27%)	
*C. neoformans*	6 (30%)	2 (22%)		4 (36%)	
*T. pallidum*	3 (15%)	1 (11%)		2 (18%)	
*M. tuberculosis*	2 (10%)	2 (22%)			
Others^c^	3 (15%)	1 (11%)		2 (18%)	
Other diagnoses	173 (50%)	153 (74%)		20 (34%)	
CNS autoimmune disorder	21 (6%)	17 (8%)		4 (7%)	
Systemic infection	68 (20%)	62 (30%)		6 (10%)	
Other neurological disease	84 (24%)	74 (36%)		10 (17%)	

*Note*: Data are shown as frequencies (%) or median (interquartile range). BM = bacterial meningitis; VZV = varicella zoster virus; HSV = Herpes simplex virus; VME = viral meningoencephalitis.

^a^ Bacterial pathogens with a single occurrence, PACEM (*S. aureus*; *S. milleri*) and MeninGene (*S. pyogenes*).

^b^ Viral pathogens with a single occurrence, PACEM (Epstein–Barr virus; human herpesvirus 7; HIV; John Cunningham virus).

^c^ Other pathogens with a single occurrence, PACEM (*T. gondii*), I‐PACE (*Leptospira*; *T. whipplei*).

In total, 88 of 343 (26%) episodes were diagnosed with bacterial meningitis, and 85 of 343 (25%) episodes had the diagnosis microbiologically confirmed in CSF. *Streptococcus pneumoniae* occurred in 50 of 85 (59%) cases, *Neisseria meningitidis* in 19 (22%), *Listeria monocytogenes* in 11 (13%), and *Haemophilus influenzae* in 2 (2%). Other bacterial pathogens were *Staphylococcus aureus*, *Streptococcus milleri*, and *Streptococcus pyogenes*, and occurred once. Viral meningoencephalitis occurred in 50 of 343 (15%) episodes, and was microbiologically confirmed by PCR in 37 (11%) episodes. An enterovirus was found in 12 of 37 (32%) cases, a Herpes simplex virus (type 1, 2, or unspecified) in 12 (32%), and a varicella zoster virus in 9 (24%). Other viral pathogens included Epstein–Barr virus, human herpesvirus 7, HIV, and John Cunningham virus, all of which occurred once. Other CNS infections were diagnosed in 32 of 343 (9%) episodes, and in 20 (6%) episodes the diagnosis was microbiologically confirmed. Pathogens included *Angiostrongylus cantonensis* in 2 of 20 (10%) cases, *Borrelia burgdorferi* in 4 (20%), *Cryptococcus neoformans* in 6 (30%), *Treponema pallidum* in 3 (15%), and *Mycobacterium tuberculosis* in 2 (10%). Other pathogens, with a single occurrence, were *Toxoplasma gondii*, *Leptospira spp*., and *Tropheryma whipplei*.

### Comparison and Clustering of CSF Metabolome between Categories

**Figure 1:**
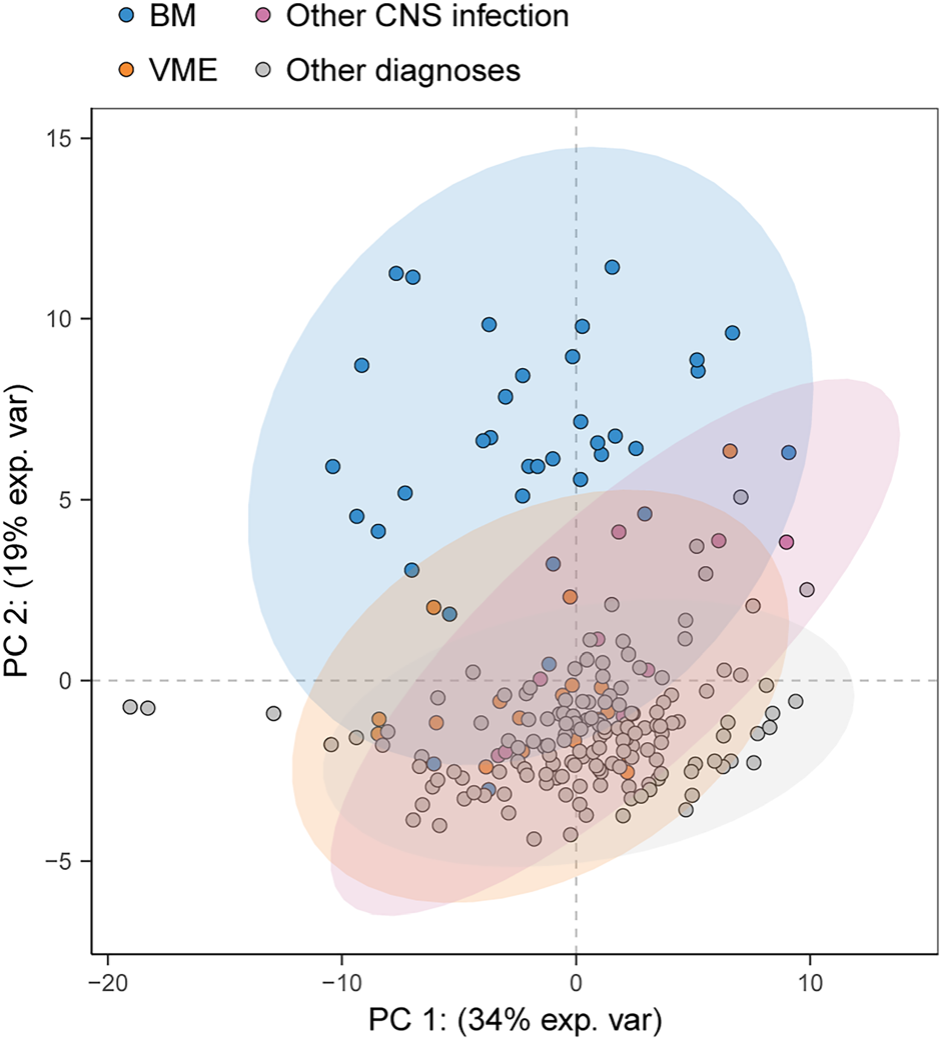
Principal component analysis of cerebrospinal fluid metabolome colored by group. Proportion of variance per component is indicated on the axis. BM = bacterial meningitis; VME = viral meningoencephalitis. [Color figure can be viewed at www.annalsofneurology.org]

**Figure 2:**
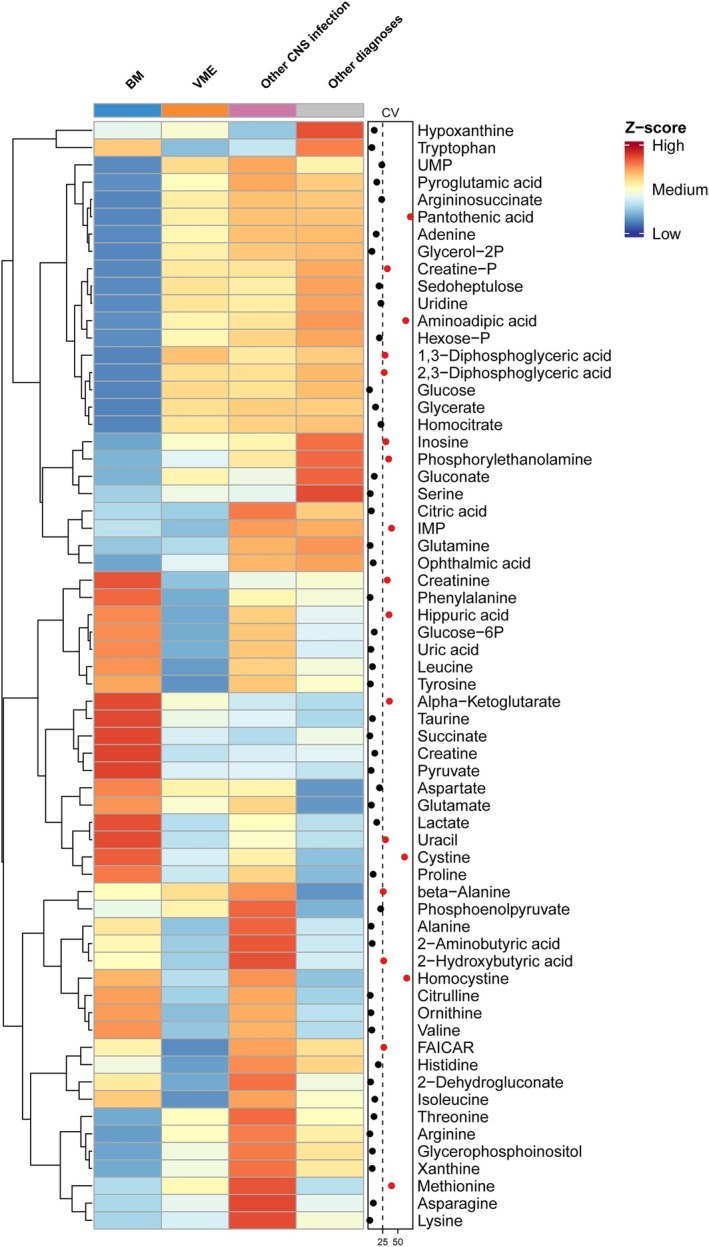
Heatmap of the average relative abundance of cerebrospinal fluid metabolites per group. Average relative abundance was calculated after log10‐transformation. Metabolites with a coefficient of variance >25% (vertical dashed line) are shown in red, but interpretation of these metabolites should be done with caution, as the variation of these metabolites is large. BM = bacterial meningitis; VME = viral meningoencephalitis. [Color figure can be viewed at www.annalsofneurology.org]

**Figure 3:**
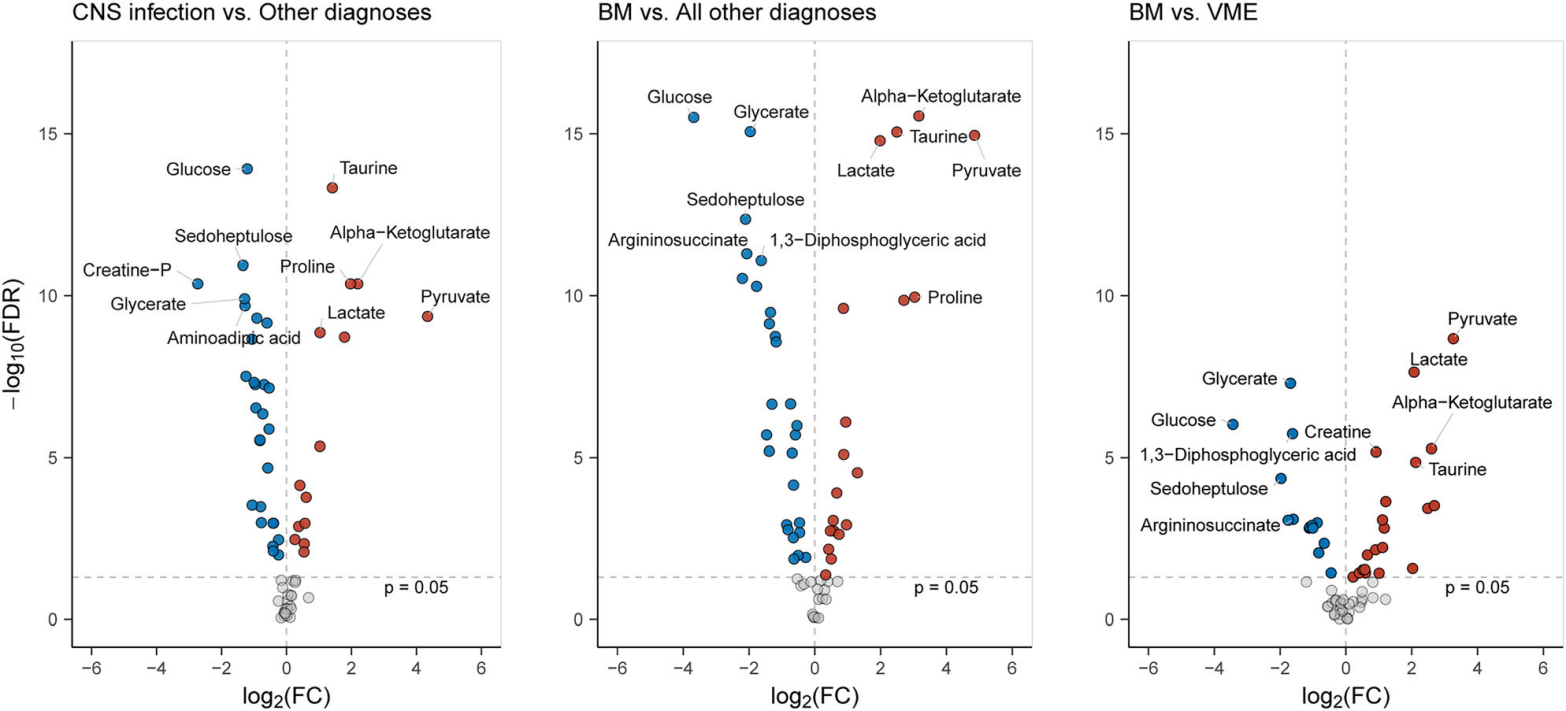
Volcano plots showing the difference in relative abundance of cerebrospinal fluid metabolites. Colors indicate direction of change. Top 5 metabolites with a FDR ≤0.05 are labeled. Metabolites showing no difference (FDR >0.05) are depicted in grey. BM = bacterial meningitis; VME = viral meningoencephalitis. [Color figure can be viewed at www.annalsofneurology.org]

A total of 64 metabolites were identified in CSF. To determine whether and how the CSF metabolome differed between all diagnostic categories, we first performed a principal component analysis (Fig. 1). Episodes with bacterial meningitis showed a distinct separation from the other groups. The average relative abundance of individual CSF metabolites per group was visualized in a heatmap (Fig. 2; Fig. S1 for the validation cohort), and 18 of 64 (28%) of metabolites had a coefficient of variance percentage of ≥25%. Significant differences in metabolite measurements were observed for 42 of 64 metabolites (66%) when comparing CNS infections with other diagnoses (Fig. 3; Table S1A). Bacterial meningitis compared with all other diagnoses yielded a significant difference for 47 of 64 (73%) metabolites, and bacterial meningitis versus viral meningoencephalitis resulted in 35 of 64 (55%) metabolites with significant differences. The same comparison, but in all episodes with a clinical diagnosis (including those without microbiological confirmation), yielded comparable results (Fig. S2A and Table S1B). The subpopulation with CSF leukocytes between 5 and 1,000 cells/mm^3^ yielded 19 of 64 (30%) significantly different metabolites for CNS infection versus other diagnoses, 27 (42%) for bacterial meningitis versus all other diagnoses, and 18 (28%) for bacterial meningitis versus viral meningoencephalitis (Fig. S2B and Table S1C).

**Figure 4:**
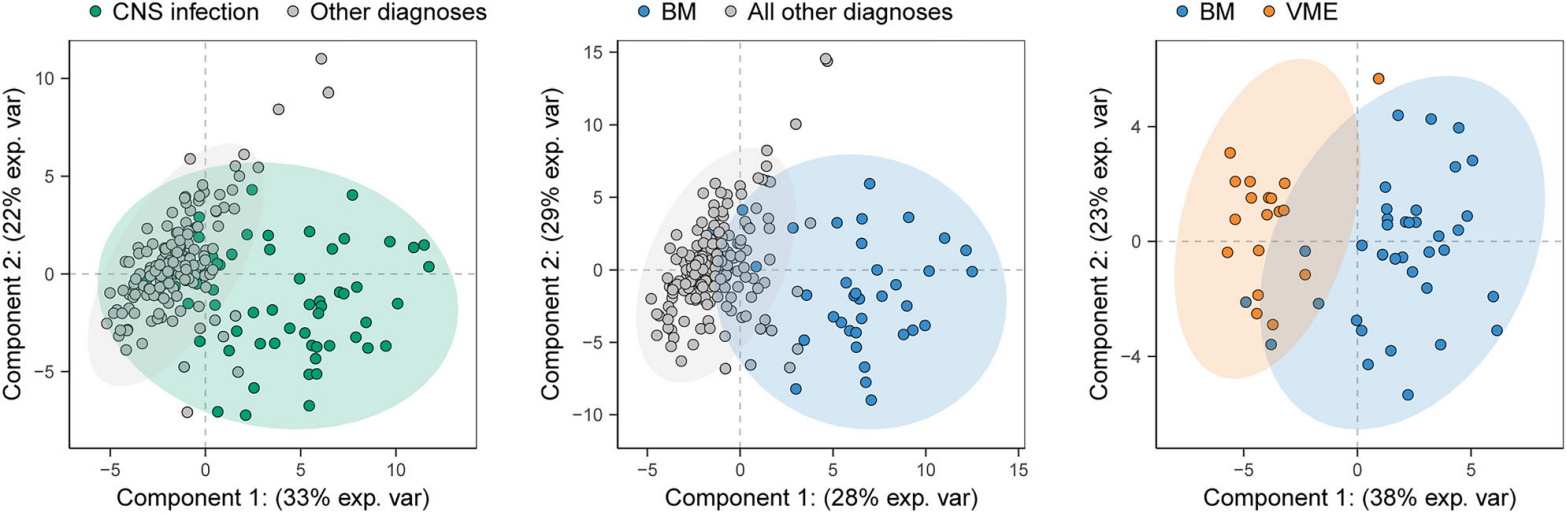
Partial least squares discriminant analysis of cerebrospinal fluid metabolome per comparison. Proportion of variance per component is indicated on the axis. BM = bacterial meningitis; VME = viral meningoencephalitis. [Color figure can be viewed at www.annalsofneurology.org]

Next, a partial least squares‐discriminant analysis on significantly altered metabolites (FDR ≤0.05) was performed to optimize the separation between categories and identify key metabolites driving the differentiation (Fig. 4 and Table S1A). Separation between CNS infection and other diagnoses was explained by 18 of 42 metabolites (43%), of which 6 were relatively higher and 12 were lower. Bacterial meningitis versus all other diagnoses was driven by 19 of 47 metabolites (40%), of which 7 were relatively higher and 12 were lower. Bacterial meningitis versus viral meningoencephalitis was driven by 13 of 35 metabolites (37%), of which 7 were relatively higher and 5 were lower. Comparable results were found in all episodes, including those without microbiological confirmation (Fig. S3A and Table S1B). In the subpopulation with CSF leukocytes between 5 and 1,000 cells/mm^3^, differentiation between CNS infection and other diagnoses was due to 8 of 19 (42%) metabolites (taurine was relatively higher; 7 were lower), bacterial meningitis and all other diagnoses due to 10 of 27 (37%) metabolites (3 were relatively higher; 7 were lower), and bacterial meningitis and viral meningoencephalitis due to 7 of 18 (39%) metabolites (4 were relatively higher; 3 were lower; Fig. S3B and Table S1C).

### Diagnostic Accuracy of CSF Metabolites

Compared with CSF metabolites, CSF leukocytes had the highest AUC when distinguishing CNS infections or bacterial meningitis from other diagnoses (AUC 0.96 [95% CI 0.93–0.99] and 0.96 [95% CI 0.94–0.99]), but not for distinguishing bacterial meningitis from viral meningoencephalitis (0.87 [95% CI 0.77–0.96]; Table 2). To distinguish CNS infections from other diagnoses, CSF metabolites showed fair to good discrimination (AUC range 0.73–0.85), with glucose (AUC 0.85 [95% CI 0.79–0.91]) and taurine (AUC 0.84 [95% CI 0.78–0.91]) being the most predictive. For bacterial meningitis versus all other diagnoses, metabolites demonstrated good to excellent discrimination (AUC range 0.82–0.95), and glucose (AUC 0.95 [95% CI 0.92–0.99]) and alpha‐ketoglutarate (AUC 0.95 [95% CI 0.92–0.99]) were among the best individual predictors. When comparing bacterial meningitis with viral meningoencephalitis, metabolites showed fair to excellent discrimination (AUC 0.79–0.97). Of those, six metabolites had an AUC higher than CSF leukocytes, which were pyruvate (AUC 0.97 [95% CI 0.93–1.00]), lactate (AUC 0.95 [95% CI 0.89–1.00]), glycerate (AUC 0.94 [95% CI 0.88–1.00]), glucose (AUC 0.91 [95% CI 0.84–0.99]), 1,3‐diphosphoglyceric acid (AUC 0.90 [95% CI 0.82–0.99]), and alpha‐ketoglutarate (AUC 0.89 [95% CI 0.78–1.00]). Similar results were found when comparing all episodes, including those without microbiological confirmation, although generally with slightly lower AUCs (Table S2A). In the subpopulation of episodes with CSF leukocytes between 5 and 1,000 cells/ mm^3^, CSF leukocytes had the highest predictive value only when comparing CNS infections with other diagnoses (AUC 0.86 [95% CI 0.80–0.93]) (Table S2B). Here, CSF metabolites showed poor to fair discrimination (AUC range 0.69–0.74). When comparing bacterial meningitis with all other diagnoses, CSF metabolites showed fair to good discrimination (AUC range 0.76–0.86), all of which were higher than CSF leukocytes. Top individual predictors were glucose (AUC 0.86 [95% CI 0.74–0.98]) and glycerate (AUC 0.86 [95% CI 0.74–0.97]), respectively. Bacterial meningitis versus viral meningoencephalitis yielded 6 metabolites with good discrimination (AUC range 0.83–0.86), all of which corresponded with the 6 best performing metabolites from the suspected and CNS proven population. CSF leukocytes were not significantly different between groups and where therefore not assessed for predictive value.

### LASSO Regression of Key CSF Metabolites

**Figure 5:**
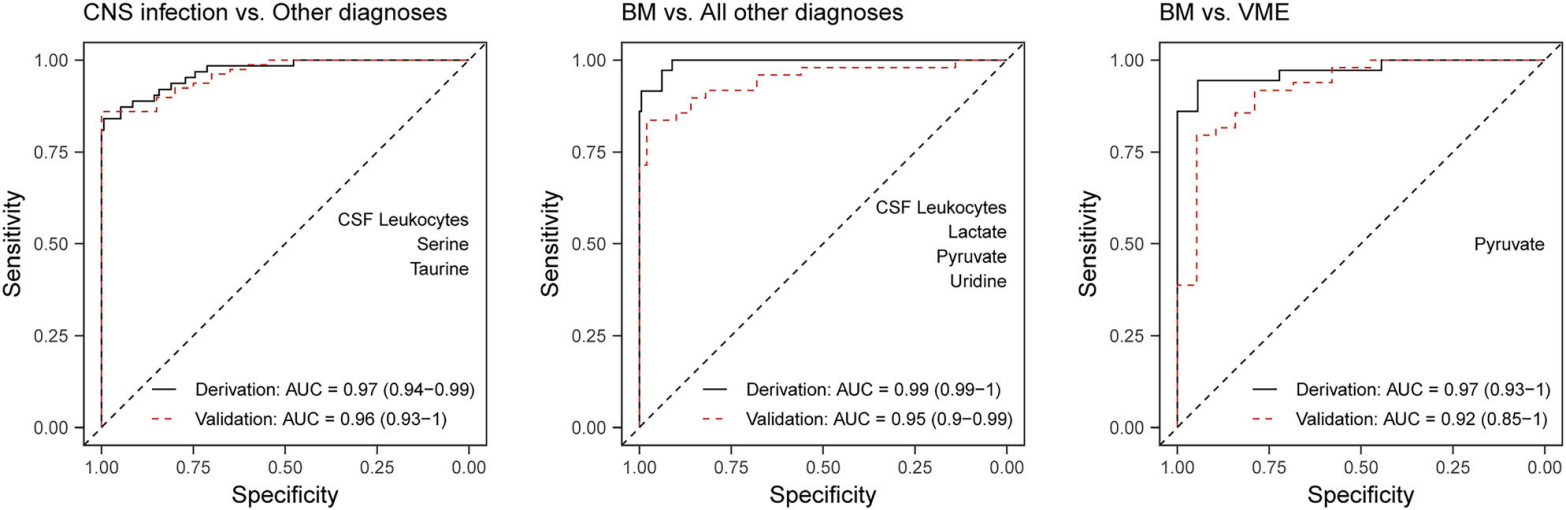
Receiver operating characteristic curves of final multivariable logistic regression models. Multivariable logistic regression models consist of variables selected in >50% of the bootstrap samples. BM = bacterial meningitis; VME = viral meningoencephalitis. [Color figure can be viewed at www.annalsofneurology.org]

To distinguish CNS infections from other diagnoses, LASSO regression in 100 bootstrap samples selected CSF leukocytes together with serine and taurine (Table S3A). The final multivariable regression model with all three variables had an AUC of 0.97 (95% CI 0.94–0.99) in the discovery cohort, and 0.96 (95% CI 0.96–1.00) in the validation cohort (Fig. 5). The Youden index (0.59) had a sensitivity and specificity of 84% and 99%, respectively (Table 3). To distinguish bacterial meningitis from all other diagnoses, the final model consisted of CSF leukocytes, lactate, pyruvate, and uridine, and demonstrated an AUC of 0.99 (95% CI 0.99–1.00) in the discovery cohort, and 0.95 (95% CI 0.90–0.99) in the validation cohort. Here, the Youden index (0.07) had 100% sensitivity and 91% specificity. Pyruvate was selected as the sole predictor to discriminate bacterial meningitis from viral meningoencephalitis, with an AUC of 0.97 (95% CI 0.93–1.00) in the discovery cohort, and 0.92 (95% CI 0.85–1.00) in the validation cohort. The Youden index (0.51) had a 94% sensitivity and 94% specificity. Comparable multivariable models with high diagnostic accuracy were found when analyzing all episodes, including those without microbiological confirmation (Fig. S4A and Table S4A). In the subpopulation of episodes with CSF leukocytes between 5 and 1,000 cells per mm^3^, the final model for CNS infection versus other diagnoses consisted of CSF leukocytes, serine, and taurine, and achieved an AUC of 0.90 (95% CI 0.84–0.96) in the discovery cohort, and 0.81 (95% CI 0.68–0.93) in the validation cohort (Fig. S4B). At a Youden index of 0.59, the model achieved 77% sensitivity and 96% specificity (Table S4B). For bacterial meningitis versus all other diagnoses, the final model consisted of lactate and uridine, and had an AUC of 0.96 (95% CI 0.93–1.00) in the discovery cohort, and 0.63 (95% CI 0.44–0.82) in the validation cohort. The Youden index was 0.30, with 87% sensitivity and 94% specificity. Glycerate and pyruvate were selected for the final model of bacterial meningitis versus viral meningoencephalitis, which had an AUC of 0.87 (95% CI 0.75–1.00) in the discovery cohort, and 0.86 (95% CI 0.71–1.00) for the validation cohort. Here, the Youden index was 0.57, and had 67% sensitivity and 100% specificity.

**Table 2:** Diagnostic Accuracy of Leukocytes and Metabolites in Cerebrospinal Fluid

Variables	CNS infection vs other diagnoses		Bacterial meningitis vs all other diagnoses		Bacterial meningitis vs viral meningoencephalitis	
	AUC	(95% CI)	AUC	(95% CI)	AUC	(95% CI)
CSF leukocytes	0.96	(0.93–0.99)	0.96	(0.94–0.99)	0.87	(0.77–0.96)
**Metabolites**						
Pyruvate	0.78	(0.72–0.85)	0.94	(0.90–0.97)	0.97	(0.93–1.00)
Alpha‐Ketoglutarate	0.80	(0.73–0.87)	0.95	(0.92–0.99)	0.89	(0.78–1.00)
Glucose	0.85	(0.79–0.91)	0.95	(0.92–0.99)	0.91	(0.84–0.99)
Lactate	0.77	(0.70–0.85)	0.94	(0.88–0.99)	0.95	(0.89–1.00)
Glycerate	0.79	(0.73–0.86)	0.94	(0.90–0.99)	0.94	(0.88–1.00)
Taurine	0.84	(0.78–0.91)	0.94	(0.89–0.99)		
Sedoheptulose	0.81	(0.75–0.88)	0.90	(0.83–0.96)	0.86	(0.76–0.96)
1.3‐Diphosphoglyceric acid	0.73	(0.65–0.81)	0.87	(0.80–0.95)	0.90	(0.82–0.99)
Argininosuccinate	0.77	(0.70–0.84)	0.88	(0.82–0.94)		
Aminoadipic acid	0.79	(0.72–0.86)	0.86	(0.79–0.93)		
Glycerol‐2P	0.78	(0.71–0.86)	0.86	(0.78–0.95)	0.80	(0.68–0.92)
Cystine	0.77	(0.70–0.84)	0.85	(0.78–0.93)		
Uridine	0.75	(0.67–0.82)	0.84	(0.76–0.91)		
2.3‐Diphosphoglyceric acid			0.83	(0.75–0.90)		
Homocitrate			0.82	(0.74–0.91)	0.79	(0.66–0.91)
Proline	0.80	(0.73–0.87)				
Serine	0.78	(0.71–0.84)				
Inosine	0.75	(0.68–0.82)				

*Note*: Only cerebrospinal fluid metabolites with a significant difference in relative abundance and a variable importance in projection score >1 are shown.

**Table 3:** Test Characteristics of Final Multivariable Logistic Regression Models

		Cut‐off value	Sens.	(95% CI)	Spec.	(95% CI)	NPV	(95% CI)	PPV	(95% CI)
CNS infection vs other diagnoses	Youden index	0.59	84	(75–92)	99	(98–100)	94	(90–97)	98	(94–100)
	100% sensitivity	0.02	100	(100–100)	48	(40–56)	100	(100–100)	44	(41–48)
	100% specificity	0.74	81	(71–90)	100	(100–100)	93	(89–96)	100	(100–100)
BM vs all other diagnoses	Youden index	0.07	100	(100–100)	91	(87–95)	100	(100–100)	69	(60–80)
	100% sensitivity	0.07	100	(100–100)	91	(87–95)	100	(100–100)	69	(60–80)
	100% specificity	0.85	86	(75–97)	100	(100–100)	97	(95–99)	100	(100–100)
BM vs VME	Youden index	0.51	94	(86–100)	94	(83–100)	89	(77–100)	97	(92–100)
	100% sensitivity	0.05	100	(100–100)	44	(22–67)	100	(100–100)	78	(72–86)
	100% specificity	0.87	86	(75–97)	100	(100–100)	78	(67–95)	100	(100–100)

Abbreviations: BM = bacterial meningitis; NPV = negative predictive value; PPV = positive predictive value; VME = viral meningoencephalitis.

## Discussion

We performed metabolomics on CSF and identified key metabolites with high diagnostic accuracy for CNS infections in general, particularly bacterial meningitis, among patients suspected of a CNS infection. Among the most significant metabolites, glucose, glycerate, and 1.3‐diphosphoglyceric acid were lower in bacterial meningitis, whereas pyruvate, lactate, taurine, and alpha‐ketoglutarate were higher. These metabolites were of particular interest in episodes with CSF leukocytes between 5 and 1,000 cells per mm^3^, as they offered greater predictive values for bacterial meningitis than CSF leukocytes, even more so when combined. In the broader study population (including episodes with and without microbiological confirmation), CSF leukocytes remained the strongest predictor for bacterial meningitis and CNS infection. However, when distinguishing bacterial meningitis from viral meningoencephalitis, CSF leukocytes had substantial lower diagnostic values, whereas CSF metabolites maintained high diagnostic accuracy. Pyruvate emerged as the best way to discriminate bacterial meningitis from viral meningoencephalitis, with no additional benefit of adding CSF leukocytes or other CSF metabolites.

Many of the differentiating metabolites suggested elevated energy metabolism, likely driven by CSF leukocytes (Fig. S5) and bacterial growth. Glucose, through glycolysis, is converted into intermediates, such as glycerate/1.3‐diphosphoglyceric acid, to ultimately produce pyruvate (Fig. S6). Pyruvate can then follow 2 metabolic pathways: it can enter the mitochondria to supply the citric acid (or tricarboxylic acid) cycle, evidenced by elevated alpha‐ketoglutarate levels, or it is fermented in the cytosol to produce lactate (when oxygen demand exceeds its supply). As such, pyruvate is an important parameter in the context of mitochondrial energy metabolism, and it is often interpreted alongside lactate and the lactate : pyruvate ratio. Previous studies on cerebral energy metabolism have found that episodes with community‐acquired bacterial meningitis frequently show a biochemical pattern indicating mitochondrial dysfunction (defined as a lactate : pyruvate ratio >30 with a normal or increased concentration of pyruvate ≥70 μM/L).^19^, ^20^ Although this might provide some insight into our current findings, the precise pathological mechanism remains unknown.

Elevated CSF lactate levels are well‐documented in bacterial meningitis.^21^ However, CSF lactate as a diagnostic marker for bacterial meningitis has certain limitation. It is also elevated in other CNS disorders; for instance, through cell decay in stroke and epilepsy, and its sensitivity is affected by antibiotic treatment prior to the lumbar puncture.^22^, ^23^ In our study, we found that CSF lactate had a high diagnostic accuracy for bacterial meningitis, which is in line with previous work.^21^ However, in contrast to pyruvate, lactate was found to only provide additional value when considered alongside CSF leukocytes, and even then the additional benefit was limited.

Our findings on tryptophan are consistent with previous literature, which reported higher levels in bacterial meningitis relative to viral CNS‐infections.^24^ However, because we also observed elevated tryptophan levels in patients with diagnoses other than CNS infections, it did not prove to be a suitable biomarker. This underlines the importance of including all patients that undergo a lumbar puncture for the workup of a CNS infection when evaluating CSF metabolites for potential biomarkers.

This study was subjected to limitations. The CSF samples were derived from 3 cohorts, and collected at different times and locations in the Netherlands. Although storage duration in the discovery cohort did not show significant differences between groups, storage duration in the validation cohort did show a significant difference for the other diagnoses category relative to VME and other CNS infections. Assessment of storage duration in the final models did not show a significant contribution. As for the processing of leftover CSF, this is performed in a uniform manner with processing laboratories being available 24 hours a day, 7 days a week. As such, we do not expect significant differences or delays in the storage procedure. The method for metabolomics was tailored to metabolites from the most fundamental metabolic pathways that were annotated with high confidence; therefore, excluding metabolites of which identification was less certain. Although this restricts the number of metabolites detected, it does allow for a more reliable interpretation of the results. So far, there is no universal or ideal method for the statistical analysis of untargeted metabolomics data. An important consideration in our approach was the ratio between the number of samples and variables before developing a multivariable regression model (at least 10 samples per predictor).

Untargeted metabolomics generates high dimensional data, increasing the risk of overfitting the prediction model. To mitigate this, we used variables reduction steps followed by LASSO regression in 100 bootstrap samples. Although this feature selection process may have discarded some potentially relevant metabolites, it improved the generalizability of the final models and reduced their complexity.

The CSF metabolome of episodes with bacterial meningitis showed clear separation from the other diagnoses. As such, the separation between episodes with a CNS infection in general and other diagnoses is likely to be biased by episodes with bacterial meningitis. Although this weakens the interpretation of metabolite alteration for CNS infections in general, it suggests that when an CNS infection is suspected, metabolites alterations in CSF are primarily indicative of a bacterial meningitis.

In contrast to plasma, where signal peaks are generally stable, metabolomics in CSF is rather challenging due to either very low or extremely high signal intensities. For instance, lactate, along with other metabolites, was less stable in the validation cohort, and resulted in a considerable lower diagnostic accuracy in models that heavily relied on it. Although this affects the validity of some metabolites, all metabolites incorporated in the final models were reliably measured in the discovery cohort. Whereas targeted methods may offer more accurate metabolite concentrations, the untargeted approach of our study does provide valuable insight into the overall metabolome of the CSF and the alterations that occur during an infection. Finally, both the discovery and validation cohort consisted of adult patients that were included in the Netherlands. Validation of our results in patients from different backgrounds and with varying pathogen exposures is needed to confirm the diagnostic potential of the metabolites in a broader population.

In conclusion, CSF metabolites demonstrate high diagnostic accuracy for CNS infections, particularly bacterial meningitis. Combining metabolites further improves the diagnostic performance, exceeding that of CSF leukocytes alone. These findings highlight the potential of cerebrospinal fluid metabolites to improve diagnostic accuracy in clinical practice.

## Author Contributions

S.S., M.B., and D.vdB. contributed to the conception and design of the study; S.S., S.O., M.vW., B.S., F.V., and the I‐PACE Study Group contributed to the acquisition and analysis of data; S.S. contributed to drafting the text or preparing the figures.

## Potential Conflicts of Interest

Nothing to report.

## Supporting information


**Data S1.** Supporting Information.

**Data S2.** Supporting Information.

## Data Availability

Data are provided within the manuscript or supplementary information files. Data protection regulations in the Netherlands do not allow for sharing of individual participant data. The anonymized dataset used during the current study is available from the corresponding author on reasonable request and can be directed to ipace@amsterdamumc.nl.

## Appendix A

I‐PACE Study Group (alphabetical order):

**Table:**

Judith Citroen	Onze Lieve Vrouwe Gasthuis
Björn M. van Geel	Noordwest Ziekenhuisgroep
Nina S. Groeneveld	Amsterdam Universitair Medisch Centrum, locatie AMC
Sebastiaan G.B. Heckenberg	Spaarne Gasthuis
Liora ter Horst	Amsterdam Universitair Medisch Centrum, locatie AMC
Korné Jellema	Haaglanden Medisch Centrum
Maartje I. Kester	Flevoziekenhuis
Joep Killestein	Amsterdam Universitair Medisch Centrum, locatie VUMC
Barry B. Mook	HagaZiekenhuis
Maarten J. Titulaer	Erasmus MC
Kiril E.B. van Veen	Alrijne Ziekenhuis
Yannick Resok	Haaglanden Medisch Centrum
Ingeborg E. van Zeggeren	Amsterdam Universitair Medisch Centrum, locatie AMC
