# Supplementary material for: Cerebrospinal Fluid Metabolome in Central Nervous System Infections: A Study of Diagnostic Accuracy
Source: Ann Neurol. 2025 Jun 17;98(4):851–63. doi: 10.1002/ana.27291 (PMC12542320; doi:10.1002/ana.27291)
Supplement: Supplementary file 1 — Data S1. Supporting Information. [file ANA-98-851-s002.pdf]

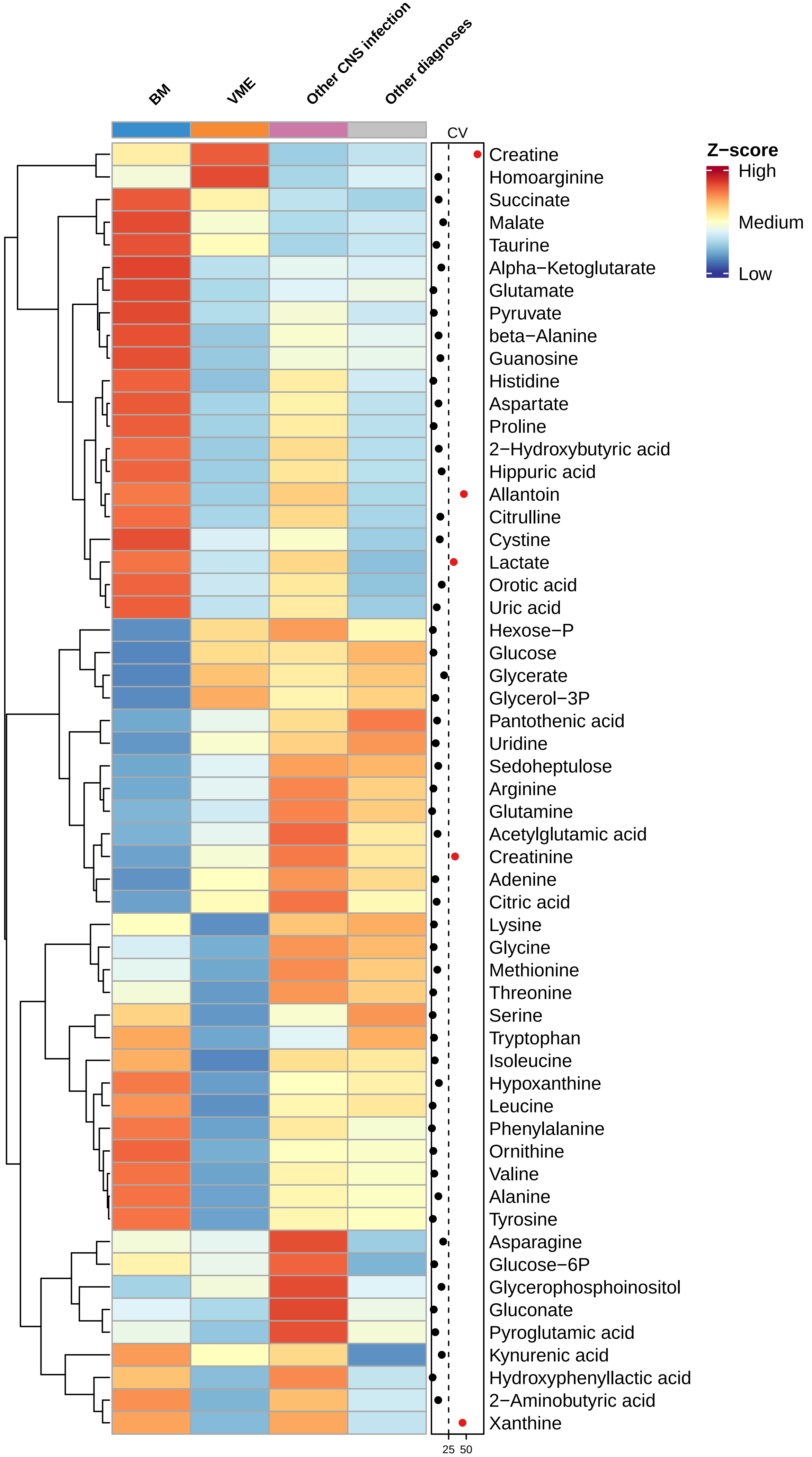

BM
VME
Other CNS infection
Other diagnoses
CV
Creatine
Homoarginine
Succinate
Malate
Taurine
Alpha−Ketoglutarate
Glutamate
Pyruvate
beta−Alanine
Guanosine
Histidine
Aspartate
Proline
2−Hydroxybutyric acid
Hippuric acid
Allantoin
Citrulline
Cystine
Lactate
Orotic acid
Uric acid
Hexose−P
Glucose
Glycerate
Glycerol−3P
Pantothenic acid
Uridine
Sedoheptulose
Arginine
Glutamine
Acetylglutamic acid
Creatinine
Adenine
Citric acid
Lysine
Glycine
Methionine
Threonine
Serine
Tryptophan
Isoleucine
Hypoxanthine
Leucine
Phenylalanine
Ornithine
Valine
Alanine
Tyrosine
Asparagine
Glucose−6P
Glycerophosphoinositol
Gluconate
Pyroglutamic acid
Kynurenic acid
Hydroxyphenyllactic acid
2−Aminobutyric acid
Xanthine
25 50
Z−score
High
Medium
Low

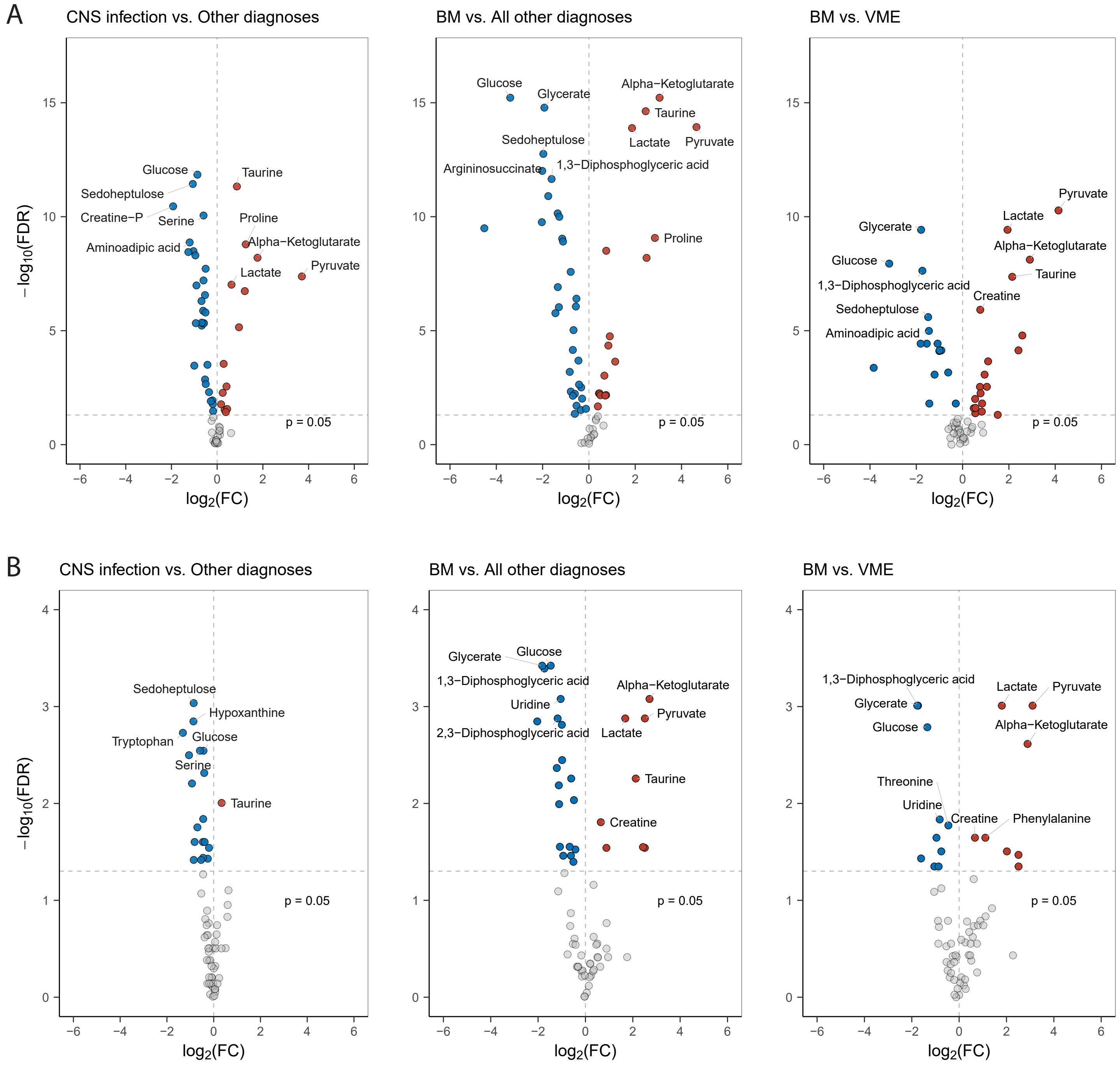
A
CNS infection vs. Other diagnoses
BM vs. All other diagnoses
BM vs. VME
Glucose
Sedoheptulose
Creatine−P
Serine
Aminoadipic acid
Taurine
Proline
Alpha−Ketoglutarate
Lactate
Pyruvate
Glycerate
Argininosuccinate
1,3−Diphosphoglyceric acid
Creatine
p = 0.05
−log10(FDR)
log2(FC)
B
CNS infection vs. Other diagnoses
BM vs. All other diagnoses
BM vs. VME
Hypoxanthine
Tryptophan
Uridine
2,3−Diphosphoglyceric acid
Threonine
Phenylalanine
p = 0.05

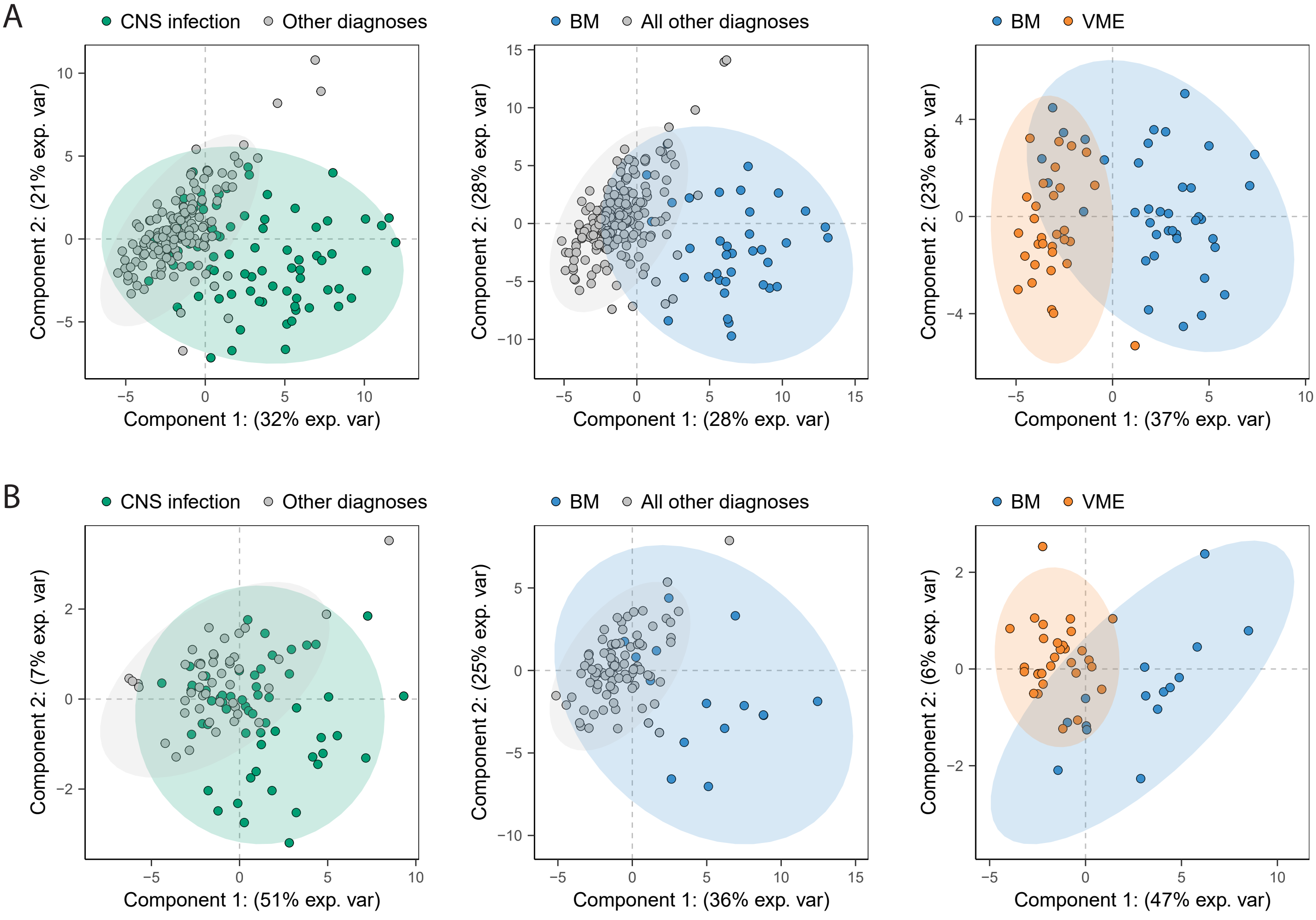

A
CNS infection
Other diagnoses
Component 1: (32% exp. var)
Component 2: (21% exp. var)
BM
All other diagnoses
Component 1: (28% exp. var)
Component 2: (28% exp. var)
BM
VME
Component 1: (37% exp. var)
Component 2: (23% exp. var)
B
CNS infection
Other diagnoses
Component 1: (51% exp. var)
Component 2: (7% exp. var)
BM
All other diagnoses
Component 1: (36% exp. var)
Component 2: (25% exp. var)
BM
VME
Component 1: (47% exp. var)
Component 2: (6% exp. var)

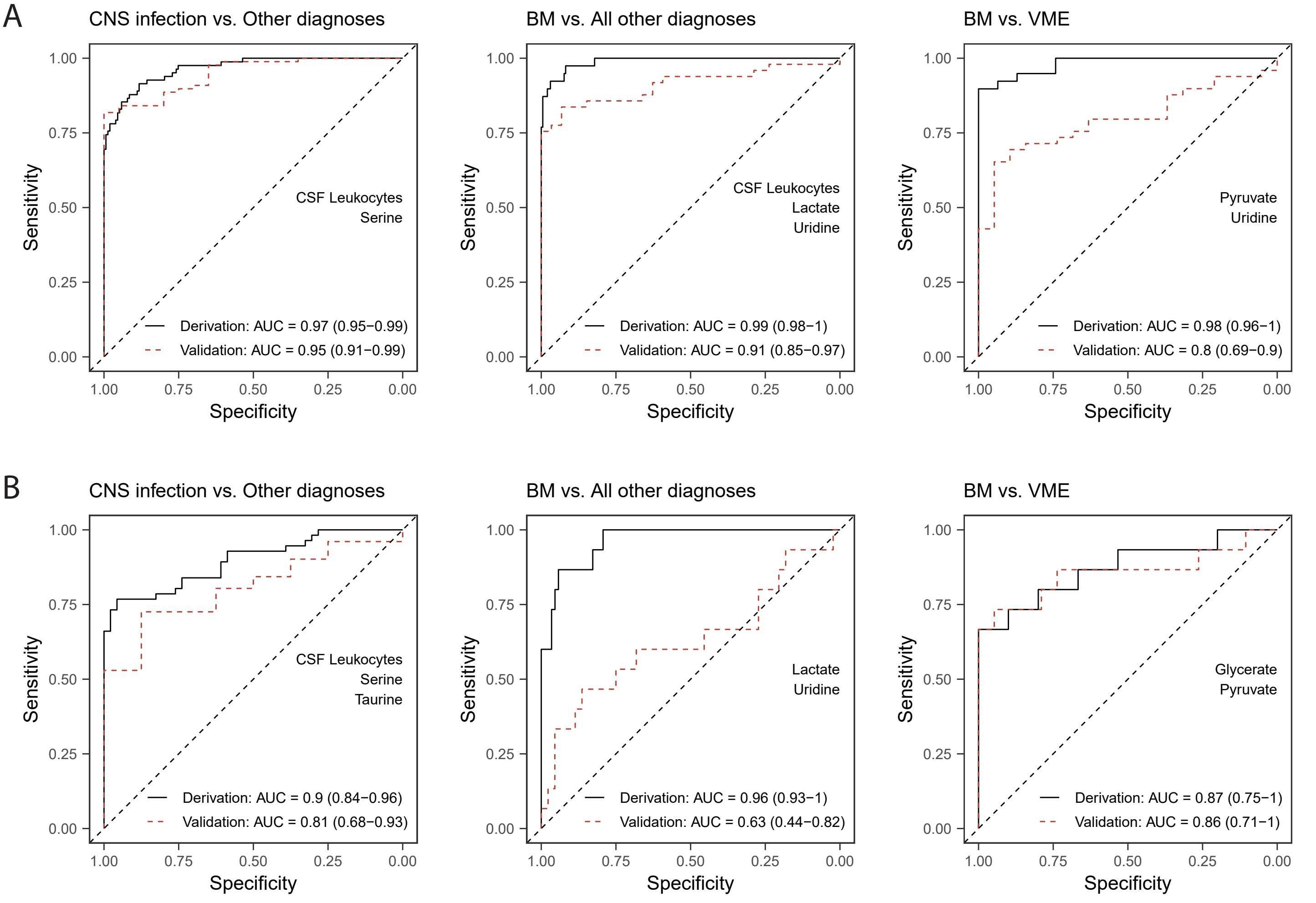
A
CNS infection vs. Other diagnoses
CSF Leukocytes
Serine
Derivation: AUC = 0.97 (0.95−0.99)
Validation: AUC = 0.95 (0.91−0.99)
BM vs. All other diagnoses
CSF Leukocytes
Lactate
Uridine
Derivation: AUC = 0.99 (0.98−1)
Validation: AUC = 0.91 (0.85−0.97)
BM vs. VME
Pyruvate
Uridine
Derivation: AUC = 0.98 (0.96−1)
Validation: AUC = 0.8 (0.69−0.9)
B
CNS infection vs. Other diagnoses
CSF Leukocytes
Serine
Taurine
Derivation: AUC = 0.9 (0.84−0.96)
Validation: AUC = 0.81 (0.68−0.93)
BM vs. All other diagnoses
Lactate
Uridine
Derivation: AUC = 0.96 (0.93−1)
Validation: AUC = 0.63 (0.44−0.82)
BM vs. VME
Glycerate
Pyruvate
Derivation: AUC = 0.87 (0.75−1)
Validation: AUC = 0.86 (0.71−1)
Sensitivity
Specificity
0.00
0.25
0.50
0.75
1.00

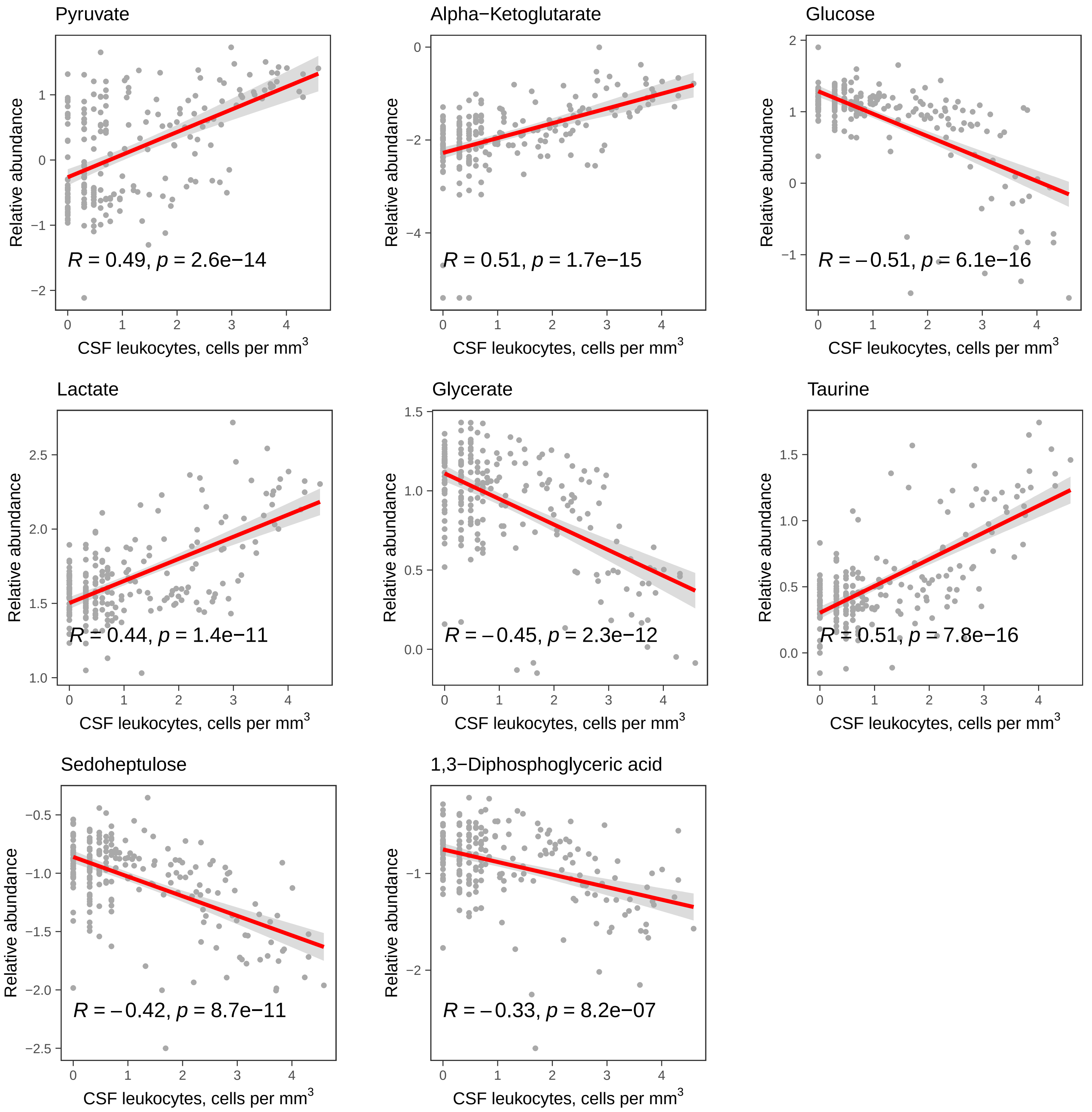
Pyruvate
R = 0.49, p = 2.6e−14
Alpha−Ketoglutarate
R = 0.51, p = 1.7e−15
Glucose
R = −0.51, p = 6.1e−16
Lactate
R = 0.44, p = 1.4e−11
Glycerate
R = −0.45, p = 2.3e−12
Taurine
R = 0.51, p = 7.8e−16
Sedoheptulose
R = −0.42, p = 8.7e−11
1,3−Diphosphoglyceric acid
R = −0.33, p = 8.2e−07
Relative abundance
CSF leukocytes, cells per mm3

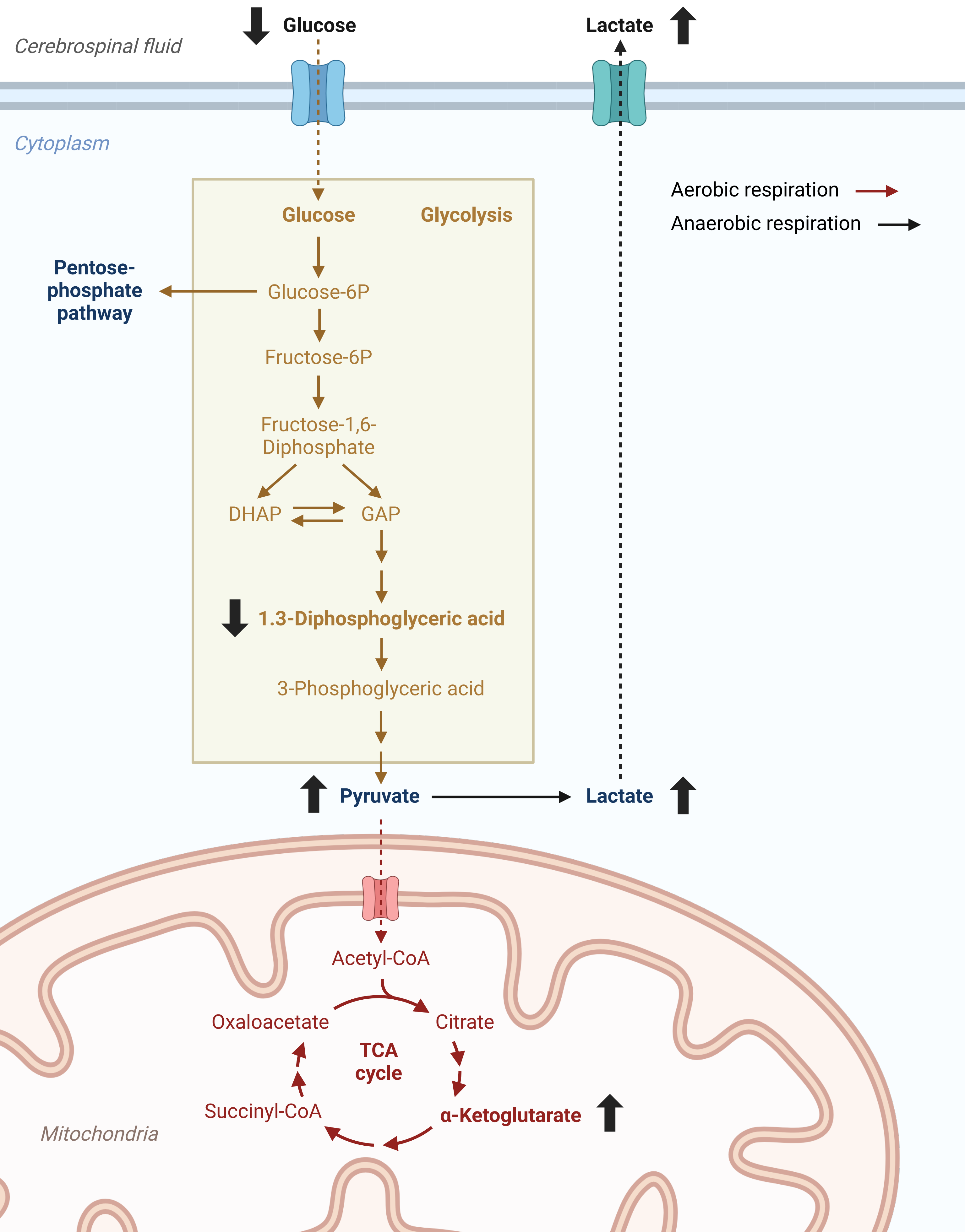
Glucose
Lactate
Cerebrospinal fluid
Cytoplasm
Aerobic respiration
Anaerobic respiration
Glucose
Glycolysis
Pentose-phosphate pathway
Glucose-6P
Fructose-6P
Fructose-1,6-Diphosphate
DHAP
GAP
1.3-Diphosphoglyceric acid
3-Phosphoglyceric acid
Pyruvate
Lactate
Acetyl-CoA
Oxaloacetate
Citrate
TCA cycle
Succinyl-CoA
α-Ketoglutarate
Mitochondria
