# Supplementary material for: Cerebrospinal Fluid Metabolome in Central Nervous System Infections: A Study of Diagnostic Accuracy
Source: Ann Neurol. 2025 Jun 17;98(4):851–63. doi: 10.1002/ana.27291 (PMC12542320; doi:10.1002/ana.27291)
Supplement: Supplementary file 2 — Data S2. Supporting Information. [file ANA-98-851-s001.zip › ana27291-sup-0002-Supinfo2.pdf]

Supplementary Table 1 Summary of comparisons per metabolite

(**A**) Episodes with a proven CNS infections. (**B**) All episodes. (**C**) Episodes with CSF leukocytes between 5-1000 cells per mm^3.

See separate Excel file.

Supplementary Table 2 Diagnostic accuracy of leukocytes and metabolites in CSF

(**A**) All episodes

	**CNS infection vs. Other diagnoses**		**Bacterial meningitis vs. All other diagnoses**		**Bacterial meningitis vs. Viral meningoencephalitis**	
Variables	AUC	(95% CI)	AUC	(95% CI)	AUC	(95% CI)
CSF leukocytes	0.96	(0.93-0.98)	0.95	(0.91-0.98)	0.85	(0.76-0.95)
**Metabolites**						
Pyruvate	0.73	(0.66-0.80)			0.94	(0.88-1.00)
Alpha-Ketoglutarate	0.74	(0.67-0.81)	0.93	(0.88-0.98)	0.90	(0.82-0.98)
Glucose	0.80	(0.74-0.86)	0.93	(0.88-0.98)	0.89	(0.82-0.97)
Lactate	0.72	(0.65-0.79)	0.91	(0.84-0.97)	0.93	(0.86-0.99)
Glycerate	0.72	(0.66-0.79)	0.92	(0.87-0.97)	0.92	(0.86-0.99)
Taurine	0.79	(0.72-0.86)	0.92	(0.86-0.98)	0.88	(0.79-0.97)
Sedoheptulose	0.79	(0.73-0.85)	0.89	(0.82-0.95)		
1.3-Diphosphoglyceric acid			0.87	(0.80-0.94)	0.89	(0.81-0.97)
Argininosuccinate	0.75	(0.68-0.81)	0.88	(0.82-0.93)		
Aminoadipic acid	0.75	(0.69-0.82)	0.86	(0.79-0.92)	0.81	(0.71-0.91)
Uridine	0.72	(0.65-0.79)	0.84	(0.77-0.91)	0.79	(0.68-0.90)
Homocitrate			0.82	(0.74-0.90)	0.79	(0.68-0.90)
2.3-Diphosphoglyceric acid			0.82	(0.75-0.89)	0.79	(0.68-0.90)
Cystine	0.72	(0.64-0.79)	0.80	(0.72-0.89)		
Serine	0.77	(0.71-0.83)				
Inosine	0.75	(0.68-0.81)	0.76	(0.67-0.84)		
Proline	0.75	(0.68-0.82)				

(**B**) Episodes with CSF leukocytes between 5-1000 cells per mm^3

	**CNS infection vs. Other diagnoses**		**Bacterial meningitis vs. All other diagnoses**		**Bacterial meningitis vs. Viral meningoencephalitis**	
Variables	AUC	(95% CI)	AUC	(95% CI)	AUC	(95% CI)
CSF leukocytes	0.86	(0.80-0.93)	0.70	(0.54-0.86)		
**Metabolites**						
Glucose	0.71	(0.61-0.81)	0.86	(0.74-0.98)	0.84	(0.7-0.98)
Glycerate			0.86	(0.74-0.97)	0.85	(0.72-0.98)
1.3-Diphosphoglyceric acid			0.85	(0.73-0.97)	0.86	(0.73-0.99)
Lactate			0.81	(0.66-0.96)	0.85	(0.72-0.99)
Pyruvate					0.85	(0.71-1.00)
Alpha-Ketoglutarate			0.83	(0.69-0.96)	0.83	(0.69-0.96)
Uridine			0.82	(0.71-0.94)		
2.3-Diphosphoglyceric acid			0.81	(0.68-0.93)		
Argininosuccinate			0.80	(0.67-0.93)		
Sedoheptulose	0.74	(0.64-0.84)	0.77	(0.63-0.91)		
Taurine	0.68	(0.58-0.79)	0.76	(0.61-0.92)		
Hypoxanthine	0.73	(0.63-0.83)				
Tryptophan	0.72	(0.62-0.82)				
Inosine	0.71	(0.61-0.81)				
Serine	0.71	(0.61-0.82)				
Aminoadipic acid	0.69	(0.59-0.80)				

Only CSF metabolites with a significant difference in relative abundance and VIP score >1 are shown.

Supplementary Table 3 Frequencies of top 10 candidate variables selected by LASSO regression

(**A**) Episodes with a proven CNS infections

Variables	**CNS infection vs. Other diagnoses** Percentage	**Bacterial meningitis vs. All other diagnoses** Percentage	**Bacterial meningitis vs. Viral meningoencephalitis** Percentage
CSF Leukocytes	**100**	**83**	19
Serine	**84**	0	0
Uridine	7	**83**	0
Pyruvate	2	**52**	**79**
Lactate	3	**73**	22
Taurine	**60**	20	0
Sedoheptulose	9	21	42
Glucose	7	33	2
Cystine	31	21	0
Glycerate	3	15	28

(**B**) All episodes

Variables	**CNS infection vs. Other diagnoses** Percentage	**Bacterial meningitis vs. All other diagnoses** Percentage	**Bacterial meningitis vs. Viral meningoencephalitis** Percentage
CSF Leukocytes	**100**	**90**	31
Uridine	7	**98**	**90**
Serine	**96**	0	0
Lactate	2	**93**	44
Pyruvate	1	0	**58**
Taurine	46	20	9
Alpha-Ketoglutarate	1	35	10
Sedoheptulose	30	5	0
Glycerate	10	9	24
Cystine	18	14	0

(**C**) Episodes with CSF leukocytes between 5-1000 cells per mm^3

Variables	**CNS infection vs. Other diagnoses** Percentage	**Bacterial meningitis vs. All other diagnoses** Percentage	**Bacterial meningitis vs. Viral meningoencephalitis** Percentage
CSF Leukocytes	**100**	33	0
Serine	**88**	0	0
Lactate	0	**88**	38
Taurine	**74**	11	0
Uridine	0	**69**	0
Glycerate	0	13	**59**
Pyruvate	0	0	**54**
Alpha-Ketoglutarate	0	46	10
Glucose	21	43	10
Sedoheptulose	32	5	0

LASSO regression followed by multivariable logistic regression analysis with backward stepwise selection was performed in 100 bootstrap samples. Variables selected in >50% of the bootstrap samples were included in the final multivariable model

Supplementary Table 4 Test characteristics of final multivariable logistic regression models

(**A**) All episodes

		Cut-off value	Sens.	(95% CI)	Spec.	(95% CI)	NPV	(95% CI)	PPV	(95% CI)
**CNS infection vs. Other diagnoses**	Youden index	0.26	91	(85-98)	88	(83-93)	95	(92-99)	81	(74-88)
	100% sensitivity	0.03	100	(100-100)	54	(46-61)	100	(100-100)	54	(50-58)
	100% specificity	0.84	70	(60-79)	100	(100-100)	86	(82-90)	100	(100-100)
**BM vs. All other diagnoses**	Youden index	0.10	97	(92-100)	92	(88-95)	99	(98-100)	71	(62-81)
	100% sensitivity	0.02	100	(100-100)	82	(77-88)	100	(100-100)	53	(46-62)
	100% specificity	0.86	77	(64-90)	100	(100-100)	96	(93-98)	100	(100-100)
**BM vs. VME**	Youden index	0.72	90	(79-97)	100	(100-100)	89	(79-97)	100	(100-100)
	100% sensitivity	0.08	100	(100-100)	74	(58-90)	100	(100-100)	83	(75-93)
	100% specificity	0.72	90	(79-97)	100	(100-100)	89	(79-97)	100	(100-100)

(**B**) Episodes with CSF leukocytes between 5-1000 cells per mm^3

		Cut-off value	Sens.	(95% CI)	Spec.	(95% CI)	NPV	(95% CI)	PPV	(95% CI)
**CNS infection vs. Other diagnoses**	Youden index	0.59	77	(64-88)	96	(89-100)	77	(69-86)	96	(89-100)
	100% sensitivity	0.11	100	(100-100)	28	(17-41)	100	(100-100)	63	(60-67)
	100% specificity	0.81	66	(54-79)	100	(100-100)	71	(64-79)	100	(100-100)
**BM vs. All other diagnoses**	Youden index	0.30	87	(67-100)	94	(89-99)	98	(94-100)	72	(56-92)
	100% sensitivity	0.05	100	(100-100)	79	(70-87)	100	(100-100)	45	(37-58)
	100% specificity	0.73	60	(33-80)	100	(100-100)	94	(90-97)	100	(100-100)
**BM vs. VME**	Youden index	0.57	67	(47-87)	100	(100-100)	86	(79-94)	100	(100-100)
	100% sensitivity	0.06	100	(100-100)	20	(7-33)	100	(100-100)	38	(35-43)
	100% specificity	0.57	67	(40-87)	100	(100-100)	86	(77-94)	100	(100-100)

BM = bacterial meningitis. VME = viral meningoencephalitis.

Supplementary Figure 1 Heatmap of the average relative abundance of CSF metabolites per group in the validation cohort.

Average relative abundance was calculated after log10-transformation. Metabolites with a CV > 25% (vertical dashed line) are also shown. but interpretation of these metabolites should be done with caution as the variation of these metabolites is large. BM = bacterial meningitis. VME = viral meningoencephalitis.

Supplementary Figure 2 Volcano plots depicting difference in relative abundance of CSF metabolites.

(**A**) All episodes. (**B**) Episodes with CSF leukocytes between 5-1000 cells per mm^3. Colors indicate direction of change. Top 5 metabolites with a FDR ≤ 0.05 are labeled. Metabolites showing no difference (FDR >0.05) are depicted in grey. BM = bacterial meningitis. VME = viral meningoencephalitis.

Supplementary Figure 3 Partial least squares discriminant analysis of CSF metabolome per comparison.

(**A**) All episodes. (**B**) Episodes with CSF leukocytes between 5-1000 cells per mm^3. Proportion of variance per component is indicated on the axis. BM = bacterial meningitis. VME = viral meningoencephalitis.

Supplementary Figure 4 ROC-curves of final multivariable logistic regression models.

(**A**) All episodes. (**B**) Episodes with CSF leukocytes between 5-1000 cells per mm^3. Multivariable logistic regression models consist of variables selected in >50% of the bootstrap samples. BM = bacterial meningitis. VME = viral meningoencephalitis.

Supplementary Figure 5 Correlation plots of the most important metabolites.

Axis are $\log_{10}$-transformed.

Supplementary Figure 6 Changes in energy metabolism in bacterial meningitis.
